# Human genital tracts microbiota: dysbiosis crucial for infertility

**DOI:** 10.1007/s40618-022-01752-3

**Published:** 2022-02-03

**Authors:** M. A. Venneri, E. Franceschini, F. Sciarra, E. Rosato, G. D’Ettorre, A. Lenzi

**Affiliations:** 1 Department of Experimental Medicine, Sapienza University of Rome, Rome, Italy; 2 Department of Public Health and Infectious Diseases, Sapienza University of Rome, Rome, Italy

**Keywords:** Microbiota, Fertility, *Lactobacillus*, Dysbiosis, Genital tracts

## Abstract

Human body is colonized by trillions of microbes, influenced by several factors, both endogenous, as hormones and circadian regulation, and exogenous as, life-style habits and nutrition. The alteration of such factors can lead to microbial dysbiosis, a phenomenon which, in turn, represents a risk factor in many different pathologies including cancer, diabetes, autoimmune and cardiovascular disease, and infertility. Female microbiota dysbiosis (vaginal, endometrial, placental) and male microbiota dysbiosis (seminal fluid) can influence the fertility, determining a detrimental impact on various conditions, as pre-term birth, neonatal illnesses, and macroscopic sperm parameters impairments. Furthermore, unprotected sexual intercourse creates a bacterial exchange between partners, and, in addition, each partner can influence the microbiota composition of partner’s reproductive tracts. This comprehensive overview of the effects of bacterial dysbiosis in both sexes and how partners might influence each other will allow for better personalization of infertility management.

## Introduction: microbial communities harbored in intestinal and genital tracts

In recent years, the existence of a human associated microbial fauna was the topic of many and very detailed studies. Scientific projects like the U.S.A. National Institutes of Health (NIS) “Human Microbiome Project” (HMP), which started in 2007 and lasted for almost a decade, provided methods and resources to detect firstly, the existence of a microbial fauna in many human body niches, and secondly, to detect microbial effects on human health [1].

Most of the human microbiota (80%) resides in the intestinal tract and is considered as an additional human organ. As a matter of fact, the gut microbiota strongly affect human health, by hindering pathogen colonization [2], exerting metabolic and trophic actions, and contributing to the development of the immune system functions [3]. As the host on which the gut microbiota is harbored is subjected to a circadian regulation, that is the influence of environmental signals deriving mainly from the day/night alternation, also the gut microbiota is subjected to its own specific circadian regulation. This regulation can influence both tropism and metabolites secretion, conditioning in turn also host homeostasis [4]. In addition, recent evidence showed how gut microbiota also exert a role in reproductive system’s development [5], influencing both male and female sexual maturation. In particular, the synthesis of metabolites, as secondary bile acids, that are gut derivatives able to influence testicular physiology [6], and of nutritional metabolites, as indole that can induce oogenesis-associated genes in some animal taxa [7] and as soybean, that has estrogenic effect [8].

Intestinal epithelial barrier harbors a large microbial community characterized by four major phyla, Bacteroidetes, Firmicutes, Actinobacteria, Proteobacteria, and an intestinal healthy microbiota composition able to maintaining intestinal homeostasis, that is characterized by > 90% of Firmicutes and Bacteroidetes, and is correlated with a low Firmicutes/ Bacteroidetes (F/B) ratio [9]. An imbalanced F/B ratio, named dysbiosis, represents a risk factor linked to many pathological consequences onset, both intestinal and whole organism related, impairing intestinal homeostasis through a microbial diversity reduction (which increases F/B ratio), and the lowering of the synthesized metabolites [9].

Only the 9% of the human microbiota was detected as harbored in urogenital tracts [10], and many studies detected their microbial composition in female and male, although the definition of their microbiota healthy state is complicated, due to the problems connected to the bacterial contamination and the detection of a low microbial concentration. In addition, numerous evidence proved how a genital microbial dysbiosis can represent a risk factor in infertility onset. In healthy conditions, females host a dominance of *Lactobacillus* bacteria, which exert many protective functions connected to lactic acid production and to the keep of vaginal acid pH [11]. Female microbiota composition was correlated to exert an influence on natural pregnancy [12] and in vitro fertilization (IVF) outcomes [13], and the variation of its constitution might be useful marker of pregnancy outcomes [14]. In male microbiota, *Lactobacillus, Pseudomonas* and *Prevotella* could exert influence on sperm quality parameters and specifically *Pseudomonas* was associated with sperm count in opposite way respect to *Prevotella,* which was instead inversely associated. Interestingly, Prevotella abundance was inversely associated with sperm concentration, and Pseudomonas was directly associated with total motile sperm count. Using a shotgun metagenomics a recent work demonstrated that the gut and urinary dysbiosis represent a risk factor for infertility, while the testicular one may have a role in mammalian spermatogenesis [15].

The aim of this review is to summarize the available findings on the most recent data about microbial colonization of the female and male genital tracts, and to highlight the features of the dysbiosis and its role on infertility.

### Interactions between gut microbiota, circadian regulation, and sexual development

The circadian regulation is the biological mechanism by the organisms living on the planet adapt their physiology and their behaviors to the astronomical day-night alternation. This system is hierarchically structured with a central clock located in the suprachiasmatic nucleus and a series of peripheral clocks present in the cells of all tissues. At the base of the mechanism there are a series of genes, called "Clock genes", [16, 17] whose regulation influence some processes like fertility and control spermatogenesis, oogenesis, steroidogenesis, pregnancy, and sexual development [18].

Gut microbiota abundance and metabolic activity presents diurnal circadian rhythm, that are mainly regulated by host nutrition and hormones. The rhythmicity gut microbiota could be loss by the disruption of clock genes highlighting a link among these factors [19]. A role of gut microbiota was observed in sexual development [20], and studies are focusing to clarify to connect gut microbiota, circadian regulation and sexual development [21]. Accumulating evidence thus suggest that gut microbiota may have an important role in determining the timing of puberty via metabolic and hormonal effects. It is also possible that gut microbiota composition is dependent on hormonal signals [22]. Interestingly, no significant microbiota discrepancies were found among the same sex infant twin pairs, however there were changes in opposite sex [23]. Gut microbiota composition and predicted metabolic profiles exist before puberty and are characteristic in male and female and become more substantial different at puberty [24]. The various species of gut microbiota changed gradually associated with puberty stages. To date, no study has examined the temporal changes of sexual dimorphism in gut microbiota in humans spanning the dynamic hormonal changes from pre-puberty to puberty. Differences in gut microflora at different pubertal status may be related to estrogen and androgen levels [20].

Growth Hormone (GH)-sexual development, implicated in gonadal development, was related to gut microbiota. Gut microbiota circadian disruption can determine GH signaling impairment, and represent a risk factor for many pathologies [25–27]. Furthermore, the GH-signaling-sexual development disruption is a common complication in pathologies linked with both circadian disruption and microbial dysbiosis, as inflammatory bowel disease (IBD) [28] and polycystic ovary syndrome (PCOS) [29].

Microbiota regulation may be influenced also by Melatonin even if the specific mechanisms are still not well understood, but it seems derived by their rhythm typically of a period of ~ 24 h regulated also by Melatonin release [30].

**Fig. 1:**
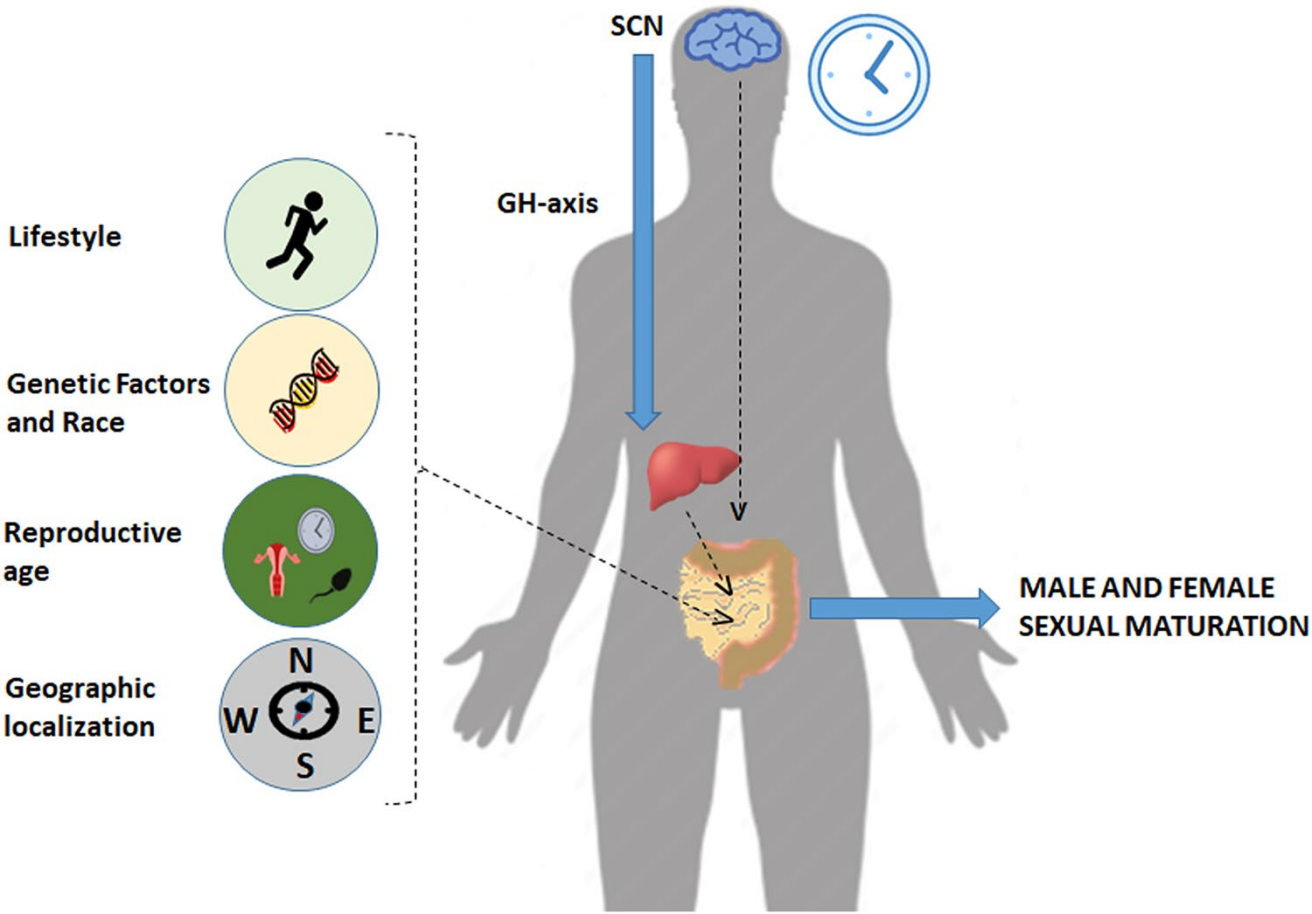
Impact of lifestyle, genetic factors, race, reproductive age, geographic localization and role of GH axis on microbiota influencing male and female sex maturation

Microbiota, circadian regulation and hormones appear to be as linked factors able to influence reciprocally, although the topic is recent, and the more studies are needed to clarify the topic Fig. 1.

### Microbial colonization in female

The balance of hosted microbiota, metabolites synthesized, and immune components is necessary for female genital tracts health. Vaginal microbiota (VMB) composition is dominated by *Lactobacillus* which represents 90–95% of vaginal bacteria. Four most abundant species detected in vaginal tract are *Lactobacillus crispatus, Lactobacillus iners, Lactobacillus jensenii* and *Lactobacillus gasseri* [31]. The importance of *Lactobacillus* and its species is linked to its ability to synthesize lactic acid (2-Hydroxypropanoic acid) by anaerobic fermentation [32], produced from vaginal epithelial cells*.* Estradiol (E2) controls lactic acid production [33] that contributes to the vaginal environment acidification (pH ≈3.0–4.5), suitable for *Lactobacillus* bacteria harboring, and enabling them to grow, multiply, and dominate the cervico-vaginal niche [34]. In addition, lactic acid is considered a healthy VMB marker, due to its mild-acid pH shift, which makes cervico-vaginal environment unsuitable for pathogenic colonization [35]. *Lactobacillus* bacteria, through lactic acid production, are able to up-regulate the autophagy process through cyclic adenosine monophosphate (cAMP) inhibition, promoting pathogen bacteria clearing. In order to kill other bacteria and to prevent vaginal colonization by pathogen species, *Lactobacillus* bacteria synthesize hydrogen peroxide (H_2_O_2_) and bacteriocins proteins [36, 37], contributing to maintain a healthy genitourinary status*.* Many other bacterial genera were detected in healthy or not-healthy vaginal microbiota, including *Actinomyces, Bacteroides, Campylobacter, Corynebacterium, Enterobacter, Escherichia, Gardnerella, Haemophilus, Salmonella, Shigella, Staphylococcus, Streptococcus, Ureaplasma* [38, 39] (Table 1).

**Table 1:** Microbiological flora of the female genital tract: colonization and action

Human fertility tracts	Harbored Bacteria		Comments	References
Cervico- vaginal tract	Phylum Firmicutes	*Lactobacillus crispatus, L.iners, L. ensenii, L. gasseri*	Lactic acid synthesis	[33]
			Hydrogen peroxide (H_2_O_2_) synthesis	[36]
			Bacteriocins peptides synthesis	[37]
			Vaginal pH acidification	
Uterine- endometrial tract	Phylum Bacteroidetes	Genus *Prevotella*	It's not possible to exclude completely microbial contamination	[42]
	Phylum Firmicutes	*L. iners, L. crispatus*		[31]
Fallopian tubes	Phylum Proteobacteria	Genera *Acinetobacter, Comamonas, Pseudomonas, Delftia*	Detected on bilateral salpingectomy surgical samples in both fertile and menopausal patients	[43]
		Genera *Pseudomonas, Burkholderia*		
	Phylum Bacteroidetes	Genus *Dysgomonas*	Detected on bilateral salpingectomy surgical samples	[43]
		Genus *Prevotella*	Detected on bilateral salpingectomy surgical samples in both fertile and menopausal patients	[43]
			Detected in left salpinx	
	Phylum Firmicutes	Genus *Vagococcus*	Detection on bilateral salpingectomy surgical samples	[43]
		Genera *Lactobacillus, Enterococcus*	Detected on left salpinx	[43]
		Genus *Staphylococcus*		
	Phylum Actinobacteria	Genus *Propionibacterium*	Detected on bilateral salpingectomy surgical samples in both fertile and menopausal patients	
Placenta	Phylum Bacteroidetes	*Prevotella tannerae*, *Bacteroides* sp.	Detected similar to oral microbiome composition	[44]
	Phylum Actinobacteria	*Streptomyces avermitilis, Propionibacterium acnes, Rhodococcus erythropolis*		
	Phylum Proteobacteria	*Neisseria polysaccharea, Neisseria lactamica*		
		*Ralstonia insidiosa, Mesorhizobum*sp.	Detected on placental villi, fetal membrane and basal plate	[45]
	Phylum Firmicutes	*Escherichia coli*	Found similar to oral microbiome composition	[44]
		*L.crispatus; L.iners; Ureaplasma nucleatum*	Detected in placental villi, fetal membrane, and basal plate	[45]
	Phylum Fusobacteria	*Fusobacterium* sp.	Detected similar to oral microbiome composition	[44]

The endometrial microbial composition detection is still controversial due to contamination, and its role remains elusive. At first, several pathogen bacterial genera like *Enterobacter*, *Gardnerella*, *Streptococcus* [40] were detected, and, in recent times, using advanced analytical techniques, Firmicutes phylum [41] and *Lactobacillus* and *Prevotella* genera were detected as the most relative abundant in hysterectomized uterus [42] (Table 1)*.*

Concerning the fallopian tubes microbial colonization, tubal microbiota (TMB) existence is a topic under study, and it was explored on bilateral salpingectomy surgical samples. In both fertile and menopausal bilateral salpingectomy patients, bacterial phyla Proteobacteria, Actinobacteria, Bacteroidetes, *Staphylococcus, Enterococcus,* and *Lactobacillus* genera were detected as the most relative abundant (Table 1) and there was detected a difference in right salpinx TMB composition (*Staphylococcus)* compared to the left one (*Lactobacillus, Enterococcus,* and *Prevotella)* [43] in pre-post-menopausal tissues. Such difference in different tubal physiological states, suggest a theoretical hormonal influence on TMB composition.

In placental samples were identified Firmicutes, Bacteroidetes, Actinobacteria, Proteobacteria, and Fusobacteria phyla (Table 1) similar to oral microbial composition [44]. Furthermore, *Mesorhizobium*, *Ralstonia, Lactobacillus* and *Ureaplasma* genera were found in placenta, although microbial composition changes in the different placental areas were examined [45].

### Role of hormone on female genital tracts microbiota

Many factors act in shaping female genital tracts microbiota composition including lifestyle, hormone, and reproductive age [18, 46]. Current studies detected E2 serum concentration as the most significant factor which determines VMB composition [47], due to its control of cervico-vaginal *Lactobacillus* dominance, and, in addition, its concentration depends on patient’s age (reproductive or menopausal), on menstrual cycle phase (follicular or luteal) and on pregnancy state. During menopause, a E2 lower concentration determines a lower glycogen and lactic acid production, with a vaginal pH shift from mild acid-to a less acid (> 4,5) environment, and a consequent loss of the *Lactobacillus* dominance [48]*.* A non-Lactobacillus-dominated environment is positively related with colonization/infection by potentially pathogenic bacteria. Postmenopausal patients, which generally have a low E2 serum concentration, show an increase of *Escherichia, Shigella, Gardnerella, Prevotella* and *Enterococcus* genera, and menopause state was also positively related with the increase of potential pathogenic genera *Varibaculum, Streptococcus,* and *Veillonella* [49]. A positive correlation between E2 concentration and the *Lactobacillus* dominance was detected in post-menopausal chronic atrophic vaginitis affected patients, however it was highlighted a negative correlation between E2 concentration and the *Streptococcus* abundance [50], a common pathogen in bacterial vaginitis [51].

The E2 levels rise during pregnancy, with an increased abundance of *Lactobacillus* and a relevant decrease in overall diversity [52]. The major changes in its compositions occur in early pregnancy, while the pregnancy later stages bacterial communities resemble those of the non-pregnant state. In the postpartum period, VMB gradually reverts to baseline characteristics, including a decrease in *Lactobacillus* abundance, an increase in diversity and enrichment of bacteria associated with vaginosis, such as *Actinobacteria* [53].

VMB composition also depends on gonadotropins, as follicle-stimulating hormone (FSH) and luteinizing hormone (LH) concentration. A negative correlation between FSH concentration and its composition in VMB was detected, and *Paraprevotella* abundance was found higher in younger patients, that have a higher FSH concentration [47]. Furthermore, a negative correlation was found between *Aerococcus*, *Atopobium,* and LH hormone concentration since their abundance is high in fertility age when the LH concentration rises [54]. On the contrary, *Gemella* was detected as positively related with both FSH and LH, because its abundance is low during fertility age and rises in menopause [55].

### Female microbiota dysbiosis

A dysbiotic VMB is characterized by the loss of *Lactobacillus* dominance and the rise of anaerobic bacteria, as *Prevotella*, *Mobiluncus*, *Gardnerella*, *Ureaplasma* and *Mycoplasma* [56, 57]. Such dysbiotic environment, represents a risk factor for the bacterial vaginosis onset, an inflammatory disease which can impair fertility in many ways: increasing sexually transmitted bacterial (*Chlamydia*, *Neisseria*, *Trichomonas*) [58] and viral (human papillomavirus (HPV) diseases, human immunodeficiency virus (HIV) infections) [59]. A dysbiotic VMB represents a risk factor also for adverse pregnancies onset, being correlated to several pregnancy related complications, like preterm delivery [60], maternal infectious morbidity [61], late miscarriage [12] and higher frequency of sudden amnio-chorial membrane rupture before labor onset (preterm premature rupture of membranes (pPROMs) [62]. VMB dysbiosis has an impact on IVF-embryo transfer outcomes: patients negative for bacterial contamination showed higher cumulative pregnancy rates, ongoing pregnancy and implantation rates [10, 63]. Interestingly, a low *Lactobacillus* abundance was identified as a predictive factor for unfavorable IVF outcome [13], while *Lactobacillus iners* as a positive IVF procedure marker and a vaginal microbiota health signal [64].

The problems connected with microbial contamination, make it hard to define precisely endometrial microbiota (EMB) composition due to *Lactobacillus* abundance. EMB dysbiosis was related to a significant decrease of IVF live birth rate positive outcomes [65].

The EMB composition related to reproductive failures was detected as also characterized by high *Bacteroides, Pelomonas* [66, 67]. However, the use of of NGS techniques permitted to detect *Flavobacterium, Corynebacterium, Bifidobacterium, Staphylococcus, Streptococcus* in infertile patients [68].

**Fig. 2:**
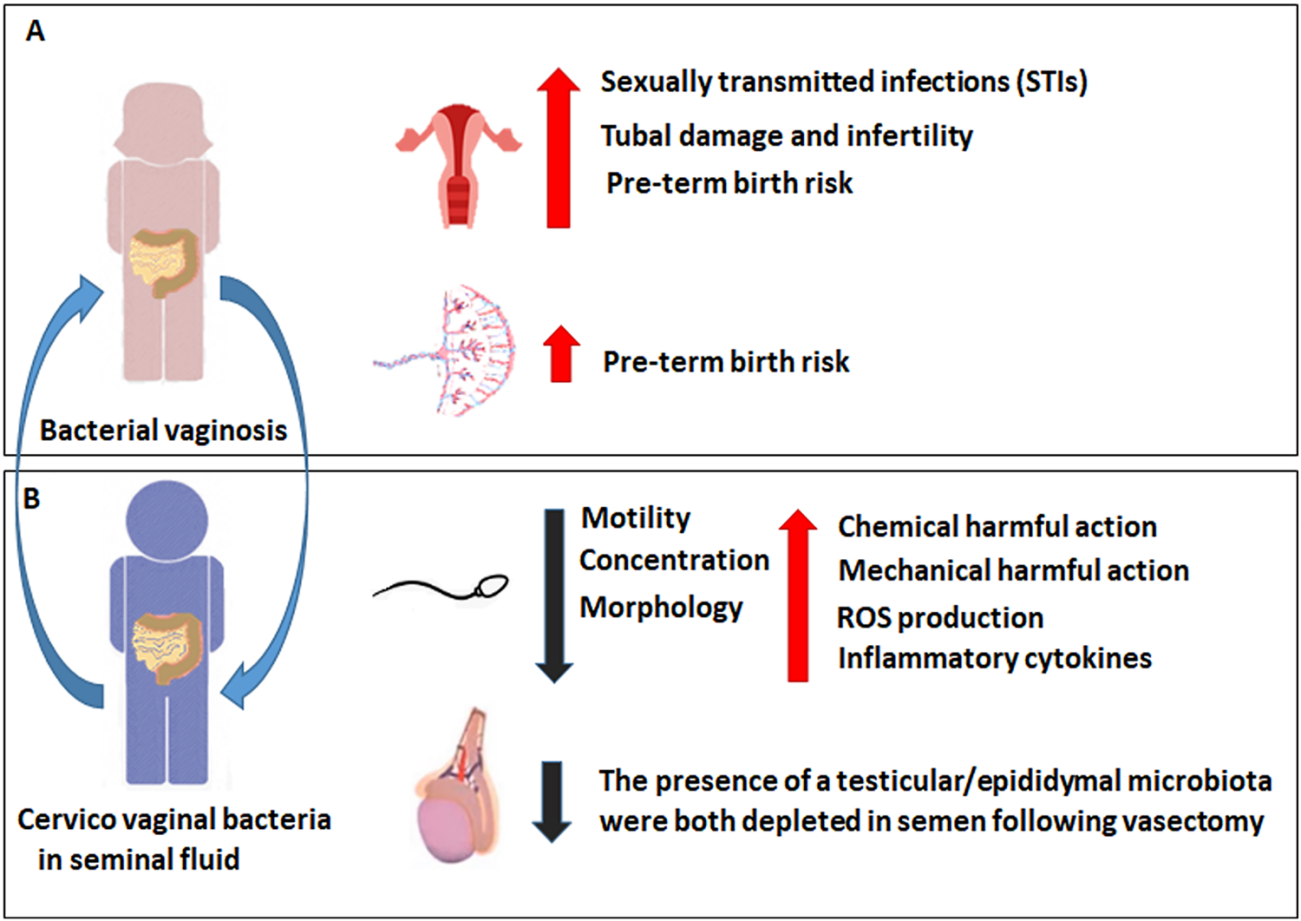
Effect of dysbiosis on female (**A**) and male (**B**) compound. Dysbiosis could influence the microbiota composition of other’s reproductive tracts. The effect of bacterial dysbiosis represents a risk factor for female fertility compartments with detrimental effects on uterus and placenta. In male dysbiosis affect macroscopic sperm parameters, with motility, morphology and concentration impairment

In placenta, the microbiota composition exerts an influence on pre-term birth or neonatal illnesses. The placental microbiota composition of spontaneous pre-term birth patients with a chorioamnionitis are characterized by high abundance of both urogenital and oral commensal bacteria [69, 70]. However, this data is controversial, because other studies on the contrary detected no microbiota composition differences in spontaneous preterm and natural birth patients (Fig. 2A).

### Microbial colonization in male

Many evidences suggest how male genitourinary tracts harbor a commensal microbial fauna, and how the seminal microbiota (SMB) have a multiple combined origin from different urogenital tissues as bladder, prostate and urethra. The seminal fluid represents a particularly suitable environment for the trophic needs of a microbial community, due to the wide range of nutrients, proteins, carbohydrates, and inorganic ions contained in its composition [71]. Both infertile and healthy patients seminal fluids show the presence of microbial bacteria, and in the seminal fluids were detected *Enterococcus faecalis*, *Escherichia coli*, *Streptococcus agalactiae*, *Ureaplasma urealyticum* species [72] (Table 2). Recently, many studies found a sperm bacterial seminal fluid composition characterized mainly by the same bacterial phyla both in healthy donors and azoospermic patients [73]. The low quality sperm morphology patients presented an augmented abundance of *Ureaplasma, Enterococcus*, *Mycoplasma,* and *Prevotella,* in opposition to healthy, which showed a major presence of *Lactobacillus* [74]. In addition, low quality sperm morphology was correlated to the presence of *Bacteroides ureolyticus* [75].

**Table 2:** Microbiological flora of the male genital tract: colonization and action

Human fertility tract	Harbored Bacteria	Comments	References
Seminal fluid	Phyla *Firmicutes;* Genera *Lactobacillus, Enterococcus, Veillonella, Streptococcus*	Bacterial phyla and species detected both in healthy donors and azoospermic patients seminal fluids	[73]
			[80]
	Phylum *Bacteroidetes*; Genus *Prevotella*,* Porphyromonas*		[73]
	Phylum *Actinobacteria*; Genus *Corynebacterium*		
	Phylum *Proteobacteria*; Genus *Acinetobacter, Pelomonas*		
	Phylum *Fusobacteria*; Genus *Sneathia*		
	Phylum *Bacteroidetes*; *Bacteroides ureolyticus*	Low quality sperm morphology marker	[73]
	Phylum *Firmicutes*; *Enterococcus faecalis*, *Escherichia coli*, *Ureaplasma urealyticum*, *Streptococcus agalactiae*	Reduced Sperm motility markers	[72]
	Phylum Bacteroidetes; Genus *Prevotella*	Low quality sperm morphology marker	[75]
	Phylum *Firmicutes*; Genus *Lactobacillus*	Seminal parameters improvement markers	

### Role of hormone on male genital tracts microbiota

Although there are no studies on the subject, it would be possible to hypothesize, based on some indirect evidence, the existence of a hormonal influence on the male genital tract microbiota. Scientific data highlighted how gut microbiota is able to communicate with host distal organs, such as the testis, determining the existence of a gut-microbiota-testis axis [76]. Although the specific mechanisms which regulate this axis have not yet been determined, it was suggest in vivo and in vitro that some endocrine disruptors compounds [77] leads to a reduction of various endocrine and steroidogenic parameters, such as serum testosterone, LH hormone, steroidogenic acute regulatory protein (StAR) expression and alter of the gut male microbiota characteristics [78]. However, more studies are needed to adequately address the matter of how endogenous hormonal influences might regulate microbiota in the male.

### Seminal microbiota dysbiosis

The detection of a causal relationship among microbial fauna presence in seminal fluid and its influence on spermatogenesis process is a topic under study for the influence on sperm quality parameters (motility, concentration, morphology), and the use of prognostic markers for dispermic conditions (oligozoospermia, astenozoospermia, teratozoospemia), azoospermia and in post-finasteride syndrome [79].

The most abundant microbial genera detected in dispermic patients and healthy donors are *Lactobacillus, Pseudomonas*, *Prevotella* and Proteobacteria, Firmicutes, Actinobacteria, Bacteroidetes, and Fusobacteria [73]. In low quality samples were detected *Prevotella*, and *Anaerococcus* [80]. *Prevotella*’s abundance was inversely associated with sperm concentration, while *Pseudomonas* was directly correlated with sperm motility [15] (Fig. 2B). *Lactobacillus* genus was significantly decreased in oligo-asthenozoospermic patients. Interestingly, different bacterial genera and species including *Ureaplasma, Bacteroides, Anaerococcus, Finegoldia, Lactobacillus* and *Acinetobacter iwoffii* could be used as asthenozoospermia biomarkers [73].

In azoospermic patients it was shown an increase of Bacteroidetes and Firmicutes, and a decrease of Proteobacteria and Actinobacteria, compared to the healthy. In the oligo-astheno-teratozoospermic (OATs) Firmicutes phylum and *Neisseria, Klebsiella, Pseudomonas* genera resulted as the most relative abundant with a decrease of *Lactobacillus* [81]. In the hyper-viscosity affected patients, Firmicutes and Proteobacteria phylum increased and *Lactobacillus* was reduced. The SMB composition of testicular tissues performed with micro testicular sperm extraction (microTESE) from idiopathic non-obstructive azoospermic (NOA) patients [82], show a decrease in *Clostridium* abundance in NOA patients with a unsuccessful sperm retrieval compared to successful sperm-retrieval patients [83].

### The effects of microbial trade-off between sexes

Unprotected sexual intercourse determines a bacterial exchange between partners, and in addition, each partner can influence the microbiota composition of other’s reproductive tracts [84]. Regarding the male influence on the female microbiota, the cervico-vaginal flora can be subjected to fluctuations after sexual intercourse, and it can represent a risk factor for bacterial vaginosis onset [85]. Moreover, sexual activity influences the composition and consistency of cervico-vaginal microbiota: it increased relevantly *Gardnerella vaginalis* in young women (with or without bacterial vaginosis), highlighting also the sexual transmission of potentially pathogenic bacteria [86]. However, bacterial sexual transmission is a difficult topic to deepen because female bacterial fluctuations are not associated only with semen microbial influence, but also with many other factors, including hormones and menstrual cycle [87].

Several evidence point also to an action of the female microbiota on male’s microbial fauna. Young men with no sexual experience showed lower bacterial diversity and relative abundance in their seminal microbiota, compared with men of the same age with sexual experience [88]. Some vaginal bacteria, as *Lactobacillus crispatus, Lactobacillus iners, Gardnerella vaginalis* resulted associated to younger men seminal microbiota, while, other genera, as, *Pseudomonas, Flavobacterium* and *Acidovorax* were detected in seminal fluid bacterial communities of older men [88, 89]. Even more, there is a correlation between male partner’s inflammatory status with leukocytes in seminal fluids, and the association with *Streptococcus agalactiae*, *Gardnerella vaginalis* and other bacterial vaginosis-related bacteria presence [90]. It was reported that *Streptococcus agalactiae* is present in bacteriospermic samples and that *Gardnerella vaginalis* is the predominant microorganism in women with partner that had significant leukocytospermia [84]. Bacterial-vaginosis episodes caused by the changing of vaginal microbial communities, was associated to frequent sexual intercourse, multiple sex partners and frequent episodes of receptive oral and anal sex [91].

Thus, scientific evidence highlighted how both male and female genital microbiota are able to interact by microbial trade off, mainly during sexual intercourses, although specific clinical trial studies are needed to clarify the topic.

## Conclusions

In recent years, great progress was made in the study of the microbial fauna associated to gut and genital tracts, detecting the existence of associated microbial communities and determining the effects of bacterial dysbiosis on fertility. Female lower genital tracts showed *Lactobacillus*-dominated healthy microbiota, and a VMB dysbiosis constitutes a risk factor for both fertility and pregnancy onset. As well, in upper genital tracts it was highlights the existence of endometrial, tubal and placental microbiota. The connections among endometrial and placental microbiota dysbiosis and fertility impairments were deepened even if in the tubal microbiota, there is no study link between dysbiosis and fertility detrimental effects. Seminal fluid dysbiosis is associated with dispermic-azoospermic detrimental conditions. The interactions among female and male genital tracts microbiota during sexual intercourse, and their microbial exchanges were strengthened, highlighting also their parallel and reciprocal effects.

Sex hormones participate in communication between microorganisms and their hosts and play several important physiological roles in reproduction and in sexual development and function.

Alterations in the genital tract microbiota have specific impacts on the reproductive endocrine system and correcting abnormal microbiomes may lead to improved reproductive outcomes. Specific linear correlations between microbiota and serum hormone levels, which may have additional effects on the health of the body, have been reported in some studies.

The knowledge of the infertility problem could be increasingly linked to the understanding of this vast network of interactions on genital tracts microbiota. Furthermore, also a greater experience of the role that the gut microbiota plays on fertility it could be useful to identify novel microbiome-based diagnostics and therapeutics for patients with this complex disease.
